# 肺腺癌细胞*EGFR*基因过表达和突变通过介导CXCR4/CXCL12信号通路表达导致肿瘤生物学行为改变的机制研究

**DOI:** 10.3779/j.issn.1009-3419.2018.07.01

**Published:** 2018-07-20

**Authors:** 佳 冯, 学燕 魏, 闯 李, 明雄 郭, 敏 彭, 启斌 宋, 光 韩

**Affiliations:** 1 430060 武汉，武汉大学人民医院肿瘤科 Department of Oncology, Renmin Hospital of Wuhan University, Wuhan 430060, China; 2 430079 武汉，湖北省肿瘤医院放疗科 Department of Radiation Oncology, Hubei Cancer Hospital, Wuhan 430079, China; 3 430072 武汉，武汉大学生命科学学院 College of Life Sciences, Wuhan University, Wuhan 430072, China

**Keywords:** EGFR, 过表达, 突变, 生物学行为, 肺肿瘤, EGFR, Overexpression, Mutations, Biological characteristics, Lung neoplasms

## Abstract

**背景与目的:**

表皮生长因子受体（epidermal growth factor receptor, *EFGR*）突变与肺腺癌侵袭转移密切相关，CXCR4/CXCL12（chemokine receptor 4/chemokine ligand 12）生物学轴在肿瘤器官特异性转移中发挥重要作用，二者在肺腺癌转移过程中是否存在相互作用尚未明确。本研究旨在探索EGFR过表达和不同位点突变对肿瘤细胞增殖、迁移和侵袭等生物学行为的影响，并探讨其潜在机制。

**方法:**

构建EGFR过表达、EGFR-E746-A750缺失型（DEL，19号外显子突变）、EGFR-T790M突变型（790M，20号外显子突变）、EGFR-L858R的突变型（LR，21号外显子突变）及EGFR空载质粒，应用Lipofectamine 2000转染H1299肺腺癌细胞。应用细胞克隆实验、细胞划痕实验和Transwell实验分别检测EGFR过表达和突变对H1299细胞增殖、迁移和侵袭能力的影响，并应用RT-PCR和Western blot检测CXCR4、CXCL12，以及该信号通路下游基因*MMP-2*和*MMP-9* mRNA和蛋白表达水平。

**结果:**

EGFR过表达和EGFR-E746-A750缺失组细胞克隆形成数目分别为28±2、28.33±4.16，且细胞迁移和侵袭能力显著高于阴性对照组与空白对照组（*P* < 0.05）。RT-PCR和Western blot实验显示EGFR过表达和EGFR-E746-A750缺失组CXCR4、CXCL12、MMP-2和MMP-9的mRNA和蛋白表达水平显著高于阴性对照组和空白对照组（*P* < 0.05）。

**结论:**

*EGFR*基因过表达和19号外显子缺失突变可通过上调CXCR4/CXCL12信号通路，促进MMP-2、MMP-9表达，从而引起肺腺癌细胞的肿瘤生物学特性发生改变，并具有更强的增殖、迁移和侵袭能力。

## Abstract (en)

**Background and objective:**

The epidermal growth factor receptor (*EFGR*) mutation was closely related to the invasion and metastasis of lung adenocarcinoma and the biological axis of CXCR4/CXCL12 (chemokine receptor 4/chemokine ligand 12) played an important role in the organ-specific metastasis of the tumor. It was a question surrounding whether there is interaction between them in the process of lung adenocarcinoma metastasis. To investigate the potential molecular mechanisms of EGFR over-expression and *EFGR*-mutations effects on cell proliferation, migration and invasion, we constructed EGFR over-expression and three *EFGR*-mutant human lung adenocarcinoma H1299 cell sublines.

**Methods:**

EGFR over-expression and three *EFGR*-mutant (EGFR-E746-A750del, EGFR-T790M and EGFR-L858R) plasmid were designed and transfected H1299 cells with Lipofectamine 2000. H1299 cells transfected with empty vector were negative control (NC), and H1299 cells without transfection were set as blank control (BC). The effects of EGFR over-expression and mutations on the proliferation, migration and invasion of H1299 cells were detected by cell cloning assay, wound healing assay and Transwell assay. The mRNA and protein expression levels of MMP-2, MMP-9, CXCR4 and CXCL12 were detected by RT-PCR and Western blot.

**Results:**

Compared with negative control group and blank control group, EGFR over-expression and EGFR-E746-A750 deletion have significantly higher colony formation (28±2, 28.33±4.16; respectively) (*P* < 0.05) and the cell migration and invasion ability were significantly increased (*P* < 0.05). RT-PCR and Western blot assay showed that the mRNA and protein expression of MMP-2, MMP-9, CXCR4 and CXCL12 in EGFR over-expression and EGFR-E746-A750 deletion group were remarkably higher than that in negative control and blank control group (*P* < 0.05).

**Conclusion:**

EGFR over-expression and 19 exon deletion can promote the expression of MMP-2 and MMP-9 by up-regulating CXCR4/CXCL12 signaling pathway, leading to the change of tumor biological characteristics such as higher proliferation, migration and invasion ability.

肺癌是目前世界上发病率和病死率最高的恶性肿瘤，其中80%-85%为非小细胞肺癌（non-small cell lung cancer, NSCLC）。研究^[1]^显示在所有NSCLC的细胞亚型中，肺腺癌患者最容易发生脑转移。同时一些回顾性临床观察研究也发现不同类型的表皮生长因子受体（epidermal growth factor receptor, *EFGR*）基因突变与肺腺癌脑转移独特的肿瘤生物学行为密切相关，是肺腺癌脑转移的一个重要的独立预测因子^[2, 3]^。然而，*EGFR*基因信号通路导致肺腺癌患者发生脑转移的具体机制尚不清楚，与其相关的基础研究也较少。CXCR4属于G蛋白偶联受体（G protein-coupled receptors, GPCRs），可选择性结合趋化因子CXCL12，构成CXCR4/CXCL12生物学轴。有研究^[4]^显示，在CXCR4/CXCL12生物学轴的趋化作用下，肺癌细胞会在脑组织中发生聚集，最终形成脑转移瘤。由此可见，CXCR4/CXCL12信号通路在肺癌细胞趋化性的向脑组织聚集的过程中发挥关键作用。那么EGFR信号通路是否是通过调节CXCR4/CXCL12信号通路的表达而导致肺腺癌细胞的具有特定的肿瘤生物学行为，使之容易向脑组织发生聚集，最终导致脑转移发生的呢？目前尚无相关研究。因此，本研究构建了EGFR过表达和3种突变肺腺癌H1299细胞株，通过观察EGFR过表达和不同位点的突变对肺腺癌H1299细胞增殖、迁移和侵袭等肿瘤生物学行为能力的影响，分析EGFR过表达和不同位点突变对CXCR4/CXCL12信号通路相关基因（CXCL12、CXCR4、MMP-2和MMP-9）表达的调节作用，以探究EGFR信号通路对肺腺癌细胞肿瘤生物学行为的影响，并探讨其潜在机制。

## 1 材料与方法

### 1.1 材料

人类H1299细胞（EGFR野生型）购自中国典型培养物保存中心（武汉大学）。RPMI-1640培养基、胎牛血清、胰酶、细胞冻存管购自ThermoFisher Scientific公司（美国）；Lipofectamine 2000、Trizol裂解液购自Invitrogen公司（美国）；RNA提取试剂盒购自ZYMO research公司（美国），TaKaRa逆转录试剂盒购自大连宝生物公司（中国），SYBR GreenPCR Master Mix（ABI）试剂盒购置ABI公司（美国）；兔源性一抗EGFR、MMP-9、CXCL12，鼠源性一抗MMP-2、CXCR4、GAPDH均购自Proteintech公司（美国）；Transwell小室购自BD Biosciences公司（美国）；不同规格的细胞培养皿和孔板购自江苏NEST生物科技有限公司，100×双抗（青霉素/链霉素）溶液购自上海碧云天生物技术有限公司。

### 1.2 方法

#### 1.2.1 细胞与培养

H1299细胞（EGFR野生型）培养于含10%胎牛血清、1%青霉素和1%链霉素的RPMI-1640培养基，置于37 ℃、5%CO_2_的培养箱中。

#### 1.2.2 载体构建与转染

EGFR过表达（OE）、EGFR-L858R的突变型（LR）、EGFR-T790M突变型（790M）、EGFR-E746-A750缺失型（DEL）及EGFR空载质粒（NC）由本实验室构建并保存。EGFR过表达和突变质粒的载体均为含GFP元件的GV358载体，克隆位点为Age Ⅰ/Age Ⅰ。*EGFR*目的基因均通过基因测序，并使用Protein Blast数据库进行序列对比分析（网址：https://blast.ncbi.nlm.nih.gov/Blast.cgi）。按照Lipofectamine 2000说明书，将上述5种质粒转染H1299细胞。转染36 h-48 h后，荧光显微镜下观察转染效率并记录保存，提取总RNA和蛋白，RT-PCR和Western blot分别检测*EGFR*基因和蛋白的表达。

#### 1.2.3 RT-PCR检测mRNA表达

以Trizol试剂提取RNA，总RNA经2%琼脂糖凝胶电泳，紫外线下观察RNA片段完整性。应用TaKaRa逆转录试剂盒将RNA逆转录成cDNA，采用SYBR GreenPCR Master Mix（ABI）试剂盒和ABI 7500 RT-PCR仪器进行分析。反应条件为：95 ℃ 10 min预变性，60 ℃ 1 min退火和延伸。采用2^-△△CT^法分析基因相对表达差异，各基因引物序列见表 1。

**1:** RT-PCR引物序列 Real-time primers

*Gene*	Primers sequences
*EGFR*	F: 5’-CTAAGATCCCGTCCATC GCC-3’
	R: 5’-GGAGCCCAGCACTTTGATCT-3’
*CXCR4*	F: 5’-ACTACACCGAGGAAATGGGCT-3’
	R: 5’-CCCACAATGCCAGTTAAGAAGA-3’
*CXCL12*	F: 5’-AAAGGCCCATTTCCTAAAAACCT-3’
	R: 5’-TGCGTTCTCTATCCAGAGGCT-3’
*MMP-2*	F: 5’-CACGCTGGGCCCTGTCACTCCT-3’
	R: 5’-TGGGGCCTCGTATACCGCATCAAT-3’
*MMP-9*	F: 5’-TGCCCGGACCAAGGATACAGTTT-3’
	R: 5’-AGGCCGTGGCTCAGGTTCAGG-3’
*GAPDH*	F: 5’-CAATGACCCCTTCATTGACC-3’
	R: 5’-GACAAGCTTCCCGTTCTCAG-3’

#### 1.2.4 Western blot法检测蛋白表达

收集转染48 h的H1299细胞，预冷PBS冲洗2次，加入蛋白裂解液，冰上裂解30 min，4 ℃ 12, 000 r/min离心10 min，应用BCA法测定总蛋白浓度。Tricine胶电泳，转膜，5%脱脂奶粉室温封闭1 h，分别孵育一抗GAPDH（1:20, 000）、MMP-2（1:1, 000）、MMP-9（1:1, 000）、CXCR4（1:6, 000）、CXCL12（1:200），4 ℃过夜，TBST洗膜6次（每次5 min）；二抗室温孵育1 h，TBST洗膜6次（每次10 min）；加入化学发光显影液，暗室中X光片曝光显影。采用Image J灰度分析软件对蛋白水平进行分析对比。

#### 1.2.5 平板克隆形成实验

将细胞接种于6孔细胞培养板中，每孔中200个单细胞，培养10 d后，弃去上清，加入4%多聚甲醛固定25 min，1%结晶紫溶液染色30 min，拍照，并统计每个6孔板中的克隆数目。实验重复3次。

#### 1.2.6 细胞划痕实验

将细胞接种于6孔细胞培养板（每孔8×10^5^个），10%胎牛血清RPMI-1640培养基（总体系2 mL），培养24 h形成单层细胞。用10 μL移液器TIP头在单层细胞上呈一字划痕，用PBS清洗3次。更换为无血清RPMI-1640培养基，分别于0 h、24 h、48 h在倒置显微镜下（×100）观察，并拍照记录。实验重复3次，结果通过Image J软件进行分析。

#### 1.2.7 Transwell实验

Transwell小室置于6孔细胞培养板，每孔加入1640培养基（不含胎牛血清）重悬的对数生长期细胞（每孔含1.5×10^4^个细胞）。下室加入500 μL含10%胎牛血清RPMI-1640培养基，置于37 ℃、5%CO_2_的培养箱中，孵育24 h。取出小室，PBS洗2遍，4%多聚甲醛室温固定25 min，1%结晶紫溶液染色30 min，应用PBS清洗。晾干后置于倒置显微镜下（×200），随机选取5个视野，统计穿过微孔膜的细胞数目并拍照记录。实验重复3次。

#### 1.2.8 统计学方法

应用SPSS 17.0软件进行统计学分析，实验组与对照组比较采用方差分析Dunnett法。*P* < 0.05为差异有统计学差异，^*^P < 0.05，^**^P < 0.01，^***^P < 0.001。

## 2 结果

### 2.1 EGFR过表达和*EGFR*突变的H1299细胞系的构建检测

转染后，EGFR过表达（OE）和EGFR-E746-A750缺失型（DEL）、EGFR-L858R的突变型（LR）、EGFR-T790M突变型（790M）和EGFR空载质粒（NC）细胞株均呈现GFP阳性，表明质粒转染成功（图 1A）。RT-PCR和Western blot检测均发现EGFR过表达和3种不同*EGFR*突变细胞中，EGFR表达均显著高于阴性对照（空载体，NC）和空白对照（H1299野生型，BC）（图 1B、图 1C）。

**1:**
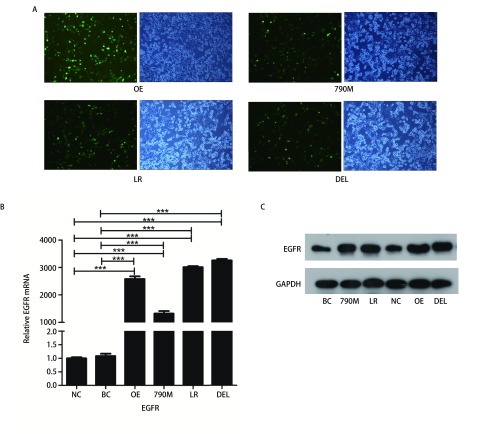
EGFR过表达和*EGFR*突变的H1299细胞系的构建检测。A：转染后，H1299细胞呈现GFP阳性（×100）；B、C：EGFR mRNA和蛋白表达情况；与阴性对照组或空白对照组相比，^***^P < 0.001。NC：阴性对照（EGFR空载体）；BC：空白对照（EGFR野生型）；790M：EGFR-T790M突变；LR: EGFR-L858R突变；OE：EGFR过表达；DEL：EGFR-E746-A750缺失突变。 Construction of EGFR over-expression and mutated H1299 cells. A: After transfection, H1299 cells showed GFP signal; B, C: Expression of EGFR mRNA and protein. Compared with negative control or blank control group, ^***^P < 0.001. NC: negative control (empty vector); BC: blank control (EGFR-wild type); 790M: EGFRT790M; LR: EGFR-L858R; OE: EGFR over-expression; DEL: EGFR-E746-A750del; EGFR: epidermal growth factor receptor.

### 2.2 EGFR过表达和EGFR-E746-A750缺失型突变促进H1299细胞增殖

EGFR过表达（OE）和EGFR-E746-A750缺失型（DEL）突变组细胞的克隆数（OE: 28±2, DEL: 28.33±4.16）显著高于阴性对照组（NC: 18±6.25）（图 2，*P* < 0.01），而EGFR-L858R和EGFR-T790M突变组与对照组克隆形成未见明显差异（图 2，*P* > 0.05）。

**2:**
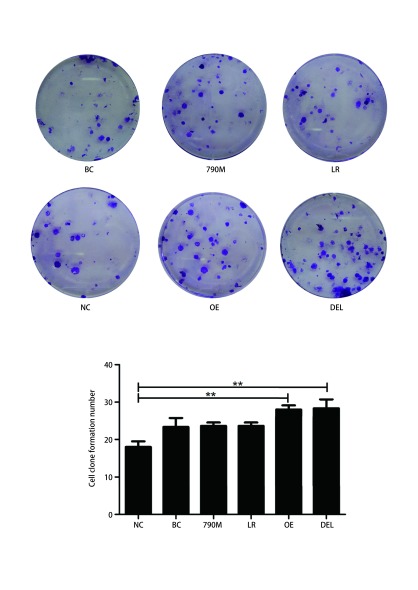
EGFR过表达和*EGFR*突变对H1299细胞增殖能力的影响。与阴性对照组或空白对照组相比，^**^P < 0.01。 Effects of EGFR over-expression and mutations on cell proliferation in H1299 cells. Compared with negative control group or blank control group, ^**^P < 0.01.

### 2.3 EGFR过表达和EGFR-E746-A750缺失型突变促进H1299细胞迁移

24 h后划痕实验结果显示，EGFR过表达（OE）和EGFR-E746-A750缺失型（DEL）突变组分别促进划痕愈合（52.62%±5.72%）、（47.60%±5.55%），且与对照组相比，差异均具有统计学意义（图 3，*P* < 0.05）。48 h后，OE和DEL组细胞划痕愈合则高达76.59%±3.80%、81.34%±2.40%，明显高于阴性对照组（59.70%±2.51%）和空白对照组（55.81%±4.49%）（图 3，*P* < 0.001）。EGFR-L858R（LR）和EGFR-T790M（790M）突变细胞亚株与阴性对照（NC）或空白对照（BC）细胞亚株对比迁移能力差异均无统计学意义（图 3，*P* > 0.05）。

**3:**
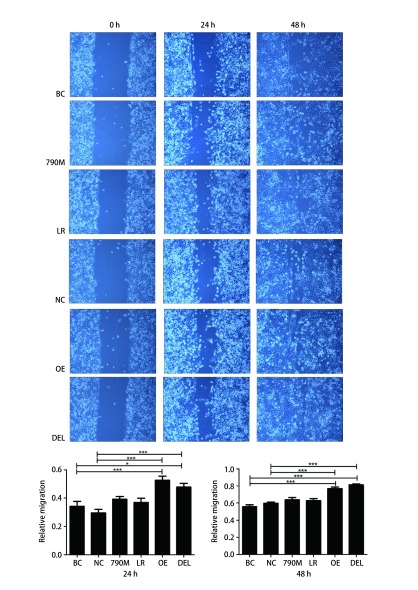
EGFR过表达和*EGFR*突变对H1299细胞迁移能力的影响。与阴性对照组或空白对照组相比，^*^P < 0.05, ^***^P < 0.001。 Effects of EGFR over-expression and mutations on cell migration in H1299 cells. Compared with negative control group or blank control group, ^*^P < 0.05, ^***^P < 0.001.

### 2.4 EGFR过表达和EGFR-E746-A750缺失型突变促进H1299细胞侵袭

Transwell小室模型检测结果显示EGFR过表达、EGFR-L858R突变和EGFR-E746-A750缺失突变组穿膜细胞数明显多于对照组（图 4，*P* < 0.001）。然而，EGFR-T790M与对照组之间无差异（图 4，*P* > 0.05）。

**4:**
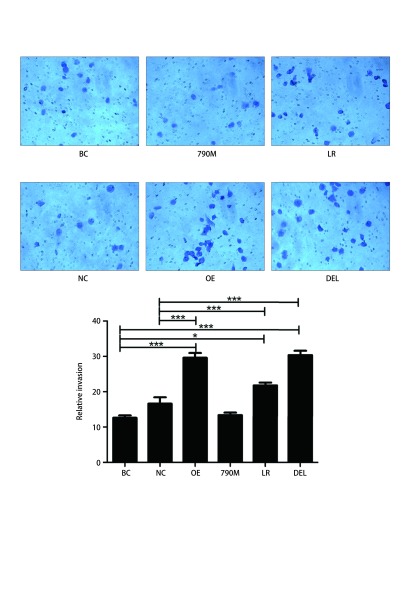
EGFR过表达和*EGFR*突变对H1299细胞侵袭能力的影响。与阴性对照组或空白对照组相比，^*^P < 0.05, ^***^P < 0.001。 Effects of EGFR over-expression and mutations on cell invasion in H1299 cells. Compared with negative control group or blank control group, ^*^P < 0.05, ^***^P < 0.001.

### 2.5 EGFR过表达和EGFR-E746-A750缺失型突变调节CXCR4、CXCL12、MMP-2和MMP-9的表达

RT-PCR进行mRNA定量，与对照组相比，结果显示EGFR过表达（OE）和EGFR-E746-A750缺失型（DEL）细胞亚株的CXCR4、CXCL12表达均升高约2倍，且差异具有统计学意义（图 5A，*P* < 0.05）。同时细胞因子MMP-2、MMP-9的mRNA表达水平在EGFR过表达（OE）和EGFR-E746-A750缺失型（DEL）细胞亚株中也均升高。Western blot实验结果进一步证实，在EGFR过表达（OE）和EGFR-E746-A750缺失型（DEL）肺癌细胞，CXCR4/CXCL12信号通路和细胞因子MMP-2、MMP-9的蛋白表达水平均显著高于对照组（图 5B）。

**5:**
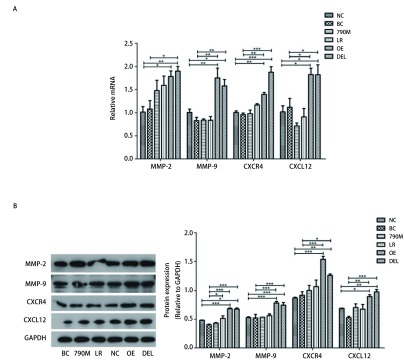
EGFR过表达和*EGFR*突变调节CXCR4、CXCL12、MMP-2和MMP-9的表达。A：CXCR4、CXCL12、MMP-2和MMP-9的mRNA表达水平；B：XCR4、CXCL12、MMP-2和MMP-9的蛋白表达情况。与阴性对照组或空白对照组相比，^*^P < 0.05, ^**^P < 0.01, ^***^P < 0.001。 EGFR over-expression and mutations regulate expression of CXCR4, CXCL12, MMP-2 and MMP-9. A: The protein expression of CXCR4, CXCL12, MMP-2 and MMP-9; B: mRNA levels of CXCR4, CXCL12, MMP-2 and MMP-9. Compared with negative control group or blank control group, ^*^P < 0.05, ^**^P < 0.01, ^***^P < 0.001.

## 3 讨论

脑是NSCLC最常见的转移器官之一，患者一旦发生脑转移，其自然生存期仅为1个月-2个月^[5]^。患者即使接受了标准的全脑放疗（whole brain radiation therapy, WBRT）后，中位生存时间也仅为3个月-6个月^[6]^。大量研究^[1, 7]^发现，在所有NSCLC的细胞亚型中，肺腺癌患者最容易发生脑转移。包括我们研究在内的既往临床回顾性研究^[8, 9]^均显示肺腺癌的*EGFR*突变和脑转移之间存在着显著相关性，且*EGFR*突变在促进脑转移的发生和发展中起了重要的作用。同时，Li等^[10]^发现在不同的*EGFR*突变的NSCLC患者中脑转移发生率存在差异。Sekine等^[11]^报道，对比野生型EGFR或EGFR 21号外显子突变的患者，19号外显子缺失的患者发生脑转移的几率更大。虽然在临床上发现了这些现象，但与其相关的基础研究甚少，且具体机制尚不清楚。如果阐明EGFR信号通路在肺腺癌患者发生脑转移中的作用机制，并对其进行预防和适宜的临床干预，那么研究EGFR信号通路在介导肺腺癌细胞特定性地向脑组织转移的肿瘤生物学特性中的作用就显得极其重要。

最经典的“种子-土壤学说”认为：脑转移的产生主要依赖于靶器官的微环境与肿瘤细胞通过细胞因子等多方面的相互作用^[12]^。有研究报道，当趋化因子CXCL12与其受体CXCR4结合可激活CXCR4/CXCL12信号通路后，该信号通路可在肿瘤细胞的增殖、粘附、迁移、侵袭、转移等生物学行为中发挥重要的作用^[13, 14]^。Phillips等^[15]^研究发现，CXCR4在NSCLC患者的肿瘤组织和多种肺癌细胞系中均高表达。Paratore等^[16]^使用定量双标记免疫荧光分析，有脑转移的原发性NSCLC患者的组织学标本中趋化因子CXCL12及其受体CXCR4的表达明显高于无脑转移者，这一发现支持了CXCR4和CXCL12在NSCLC转移扩散到脑组织过程中的作用。由此可见，CXCR4/CXCL12信号通路在肺癌细胞趋化性地向脑组织聚集的过程中发挥了重要的作用。基质金属蛋白酶（matrix metalloproteinase, MMPs）是多因子锌依赖性内肽酶，在肿瘤侵袭和转移过程中发挥关键作用^[17, 18]^。Singh等^[19]^在前列腺癌模型的研究中，已经证明了CXCR4-CXCL12相互作用可调节MMPs的表达。在MMPs中，有研究^[20-22]^发现MMP-2和MMP-9对促进NSCLC细胞发生侵袭和转移起到了重要的作用。此外，有研究^[20, 23]^表明，CXCL12可通过与CXCR4相互作用增加MMP-2、MMP-9的表达，从而促进肺癌转移。那么EGFR信号通路是否能影响上述信号通路的信号传导呢？已有研究显示在乳腺癌中EFGR信号通路可调节CXCR4/CXCL12信号通路的表达，导致转移率的提高^[24]^。在肺腺癌细胞中EGFR-L858R突变可通过激活ERK信号通路诱导CXCR4表达，从而促进癌细胞侵袭能力和恶性胸腔积液形成^[25]^。目前，尚无研究将EGFR信号通路与CXCR4/CXCL12信号通路和肺腺癌脑转移联系起来进行综合分析。本研究发现，相较于野生型，当EGFR过表达或出现19号外显子缺失突变时，肺腺癌细胞的肿瘤生物学特性会发生明显的改变，如更强的增殖、迁移和侵袭能力，同时对*MMP-2*、*MMP-9*和*CXCR4*、*CXCL12*等基因的mRNA和蛋白表达表现出明显的上调作用。这些研究结果提示EGFR过表达及其突变可以改变肺腺癌细胞肿瘤生物学特性，其定向转移的生物学特性可能是通过调节CXCR4/CXCL12信号通路的表达来实现的。

虽然本研究发现了EGFR过表达或者不同位点突变确实可以导致肺腺癌细胞发生肿瘤生物学特性的改变，尤其是19号缺失突变的细胞增殖、侵袭和转移能力明显增强，这与临床上观察到19号缺失突变的患者更容易发生脑转移的现象也相互吻合^[11]^，但由于本研究只是进行了体外细胞学实验，能否在体内复杂的环境中重复出一致的结果尚无法肯定。因此，我们将在后续的动物模型中对本研究结果加以证实。

综上所述，本研究发现*EGFR*基因过表达和19号外显子缺失突变可以通过上调CXCR4/CXCL12信号通路，促进MMP-2、MMP-9的表达，从而引起肺腺癌细胞的肿瘤生物学特性发生改变。这可能对今后临床上更精准地筛选NSCLC脑转移发生的高危人群并进行监控和预防，研制靶点作用于CXCR4/CXCL12信号通路的药物提供了坚实的基础，同时也指明了今后研究的方向。

## References

[1] Mujoomdar A, Austin JH, Malhotra R (2007). Clinical predictors of metastatic disease to the brain from non-small cell lung carcinoma: primary tumor size, cell type, and lymph node metastases. Radiology.

[2] Gow CH, Chien CR, Chang YL (2008). Radiotherapy in lung adenocarcinoma with brain metastases: effects of activating epidermal growth factor receptor mutations on clinical response. Clin Cancer Res.

[3] Shimato S, Mitsudomi T, Kosaka T (2006). *EGFR* mutations in patients with brain metastases from lung cancer: association with the efficacy of gefitinib. Neuro Oncol.

[4] Salmaggi A, Maderna E, Calatozzolo C (2009). CXCL12, CXCR4 and CXCR7 expression in brain metastases. Cancer Biol Ther.

[5] Park HS, Decker RH, Wilson LD (2015). Prophylactic cranial irradiation for Patients with locally advanced non-small-cell lung cancer at high risk for brain metastases. Clin Lung Cancer.

[6] Mehta MP, Rodrigus P, Terhaard CH (2003). Survival and neurologic outcomes in a randomized trial of motexafin gadolinium and whole-brain radiation therapy in brain metastases. J Clin Oncol.

[7] Mendelsohn J, Baselga J (2003). Status of epidermal growth factor receptor antagonists in the biology and treatment of cancer. J Clin Oncol.

[8] Shin DY, Na Ⅱ, Kim CH (2014). *EGFR* mutation and brain metastasis in pulmonary adenocarcinomas. J Thorac Oncol.

[9] Han G, Bi J, Tan W (2016). A retrospective analysis in patients with *EGFR*-mutant lung adenocarcinoma: is *EGFR* mutation associated with a higher incidence of brain metastasis?. Oncotarget.

[10] Li B, Sun SZ, Yang M (2015). The correlation between *EGFR* mutation status and the risk of brain metastasis in patients with lung adenocarcinoma. J Neurooncol.

[11] Sekine A, Kato T, Hagiwara E (2012). Metastatic brain tumors from non-small cell lung cancer with *EGFR* mutations: distinguishing influence of exon 19 deletion on radiographic features. Lung Cancer.

[12] Teicher BA, Fricker SP (2010). CXCL12 (SDF-1)/CXCR4 pathway in cancer. Clin. Cancer Res.

[13] Sun YX, Schneider A, Jung Y (2005). Skeletal localization and neutralization of the SDF-1 (CXCL12)/CXCR4 axis blocks prostate cancer metastasis and growth in osseous sites *in vivo*. J Bone Miner Res.

[14] Domanska UM, Kruizinga RC, Nagengast WB (2013). A review on CXCR4/ CXCL12 axis in oncology: No place to hide. Eur J Cancer.

[15] Phillips RJ, Burdick MD, Lutz M (2003). The stromal derived factor-1/CXCL12-CXC chemokine receptor 4 biological axis in non-small cell lung cancer metastases. Am J Respir Crit Care Med.

[16] Paratore S, Banna GL, D'Arrigo M (2011-2012). CXCR4 and CXCL12 immunoreactivities differentiate primary non-small-cell lung cancer with or without brain metastases. Cancer Biomark.

[17] Deryugina EI, Quigley JP (2006). Matrix metalloproteinases and tumor metastasis. Cancer Metastasis Rev.

[18] Ii M, Yamamoto H, Adachi Y, Maruyama Y (2006). Role of matrix metalloproteinase-7 (matrilysin) in human cancer invasion, apoptosis, growth, and angiogenesis. Exp Biol Med (Maywood).

[19] Singh S, Singh UP, Grizzle WE (2004). CXCL12-CXCR4 interactions modulate prostate cancer cell migration, metalloproteinase expression and invasion. Lab Invest.

[20] Tang CH, Tan TW, Fu WM (2008). Involvement of matrix metalloproteinase-9 in stromal cell-derived factor-1/CXCR4 pathway of lung cancer metastasis. Carcinogenesis.

[21] Chakrabarti S, Patel KD (2005). Matrix metalloproteinase-2 (MMP-2) and MMP-9 in pulmonary pathology. Exp Lung Res.

[22] Iijima T, Minami Y, Nakamura N (2004). MMP-2 activation and stepwise progression of pulmonary adenocarcinoma: analysis of MMP-2 and MMP-9 with gelatin zymography. Pathol Int.

[23] Ghosh MC, Makena PS, Gorantla V (2012). CXCR4 regulates migration of lung alveolar epithelial cells through activation of Rac1 and matrix metalloproteinase-2. Am J Physiol Lung Cell Mol Physiol.

[24] Zhang XH, Wang Q, Gerald W (2009). Latent bone metastasis in breast cancer tied to Src-dependent survival signals. Cancer Cell.

[25] Tsai MF, Chang TH, Wu SG (2015). EGFR-L858R mutant enhances lung adenocarcinoma cell invasive ability and promotes malignant pleural effusion formation through activation of the CXCL12-CXCR4 pathway. Sci Rep.

